# Supplementary material for: Ventral striatal islands of Calleja neurons bidirectionally mediate depression-like behaviors in mice
Source: Nat Commun. 2023 Oct 28;14:6887. doi: 10.1038/s41467-023-42662-z (PMC10613228; doi:10.1038/s41467-023-42662-z)
Supplement: Supplementary file 1 — Supplementary Information [file 41467_2023_42662_MOESM1_ESM.pdf]

Supplementary Information for

Ventral striatal islands of Calleja neurons bidirectionally mediate depression-like behaviors in mice

Yun-Feng Zhang[1,2,3*], Jialiang Wu[1,2#], Yingqi Wang[3#], Natalie L. Johnson[4#], Janardhan P. Bhattarai[3#], Guanqing Li[1,2,5], Wenqiang Wang[1,2,5], Camilo Guevara Espitia[3], Hannah Shoenhard[3], Marc V. Fuccillo[3], Daniel W. Wesson[4], Minghong Ma[3*]

1. State Key Laboratory of Integrated Management of Pest Insects and Rodents, Institute of Zoology, Chinese Academy of Sciences, Beijing 100101, China

2. CAS Center for Excellence in Biotic Interactions, University of Chinese Academy of Sciences, Beijing 100101, China

3. Department of Neuroscience, University of Pennsylvania Perelman School of Medicine, Philadelphia, PA 19104, USA

4. Department of Pharmacology and Therapeutics, University of Florida, Gainesville, FL 32610, USA

5. College of Life Sciences, Hebei University, Baoding, Hebei 071002, China

*Correspondence to: Yun-Feng Zhang (yfzhang@ioz.ac.cn) or Minghong Ma (minghong@pennmedicine.upenn.edu).

# These authors contributed equally.

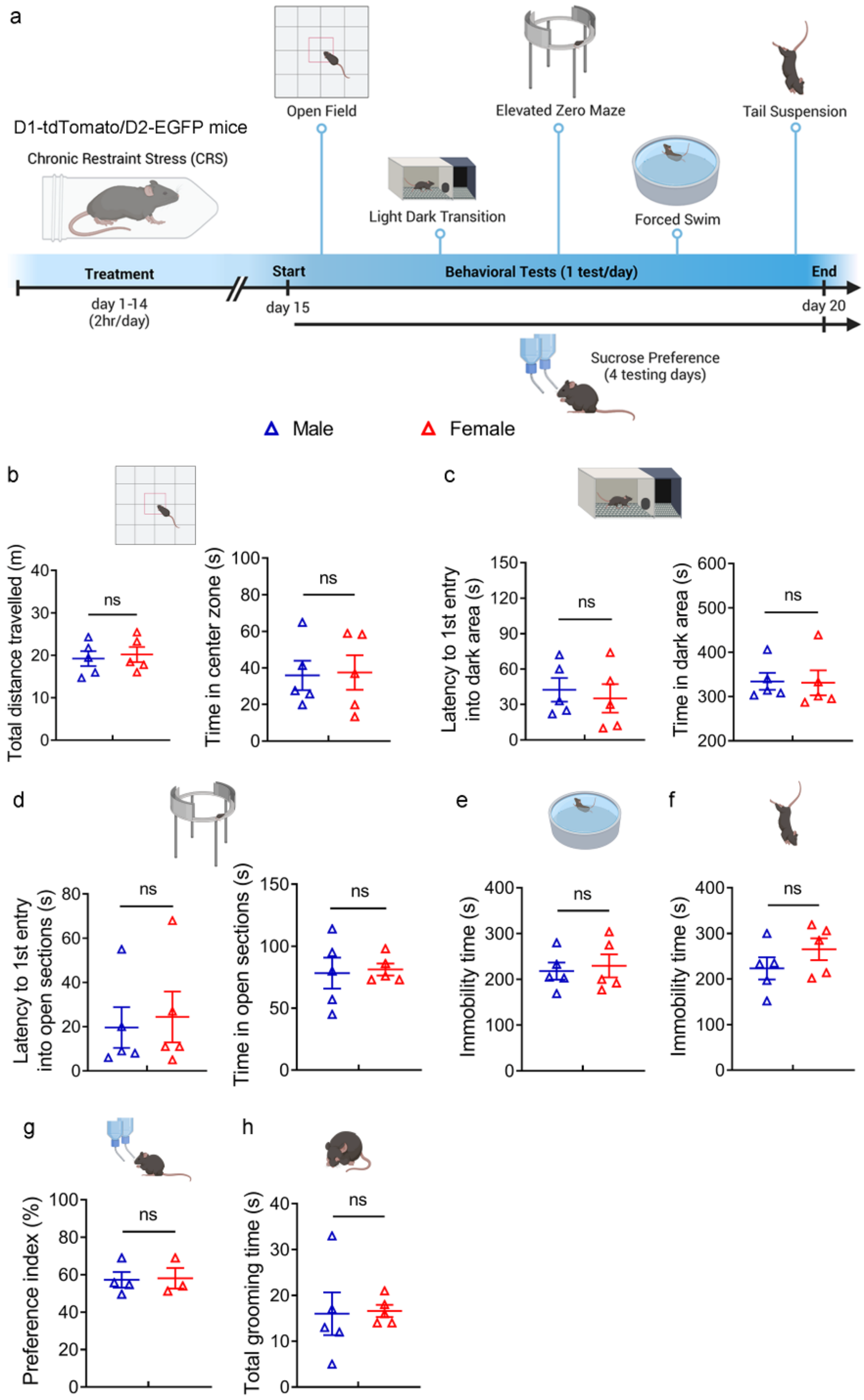

Supplementary Figure 1. Chronic restraint stress (CRS) does not induce sexually dimorphic effects on affective behaviors in D1-tdTomato/D2-EGFP mice. Related to Figure 1. (a) Experimental strategy and timeline for behavioral assays. Created with BioRender.com. (b-h) Effects of CRS on behavioral performance in different tests. (b) Open field test: total distance travelled (left; $t_{(8)}$ = 0.379 and p = 1.000) and time in the center zone (right; $t_{(8)}$ = 0.127 and p = 1.000). (c) Light-dark box transition test: latency to the first entry into the dark area (left; $t_{(8)}$ = 0.459 and p = 1.000) and time spent in the dark area (right; $t_{(8)}$ = 0.094 and p = 1.000). (d) Elevated zero maze test: latency to the first entry into open sections (left; $t_{(8)}$ = 0.326 and p = 1.000) and time in open sections (right; $t_{(8)}$ = 0.224 and p = 1.000). (e) Forced swimming test: immobility time ($t_{(8)}$ = 0.371 and p = 0.720). (f) Tail suspension test: immobility time ($t_{(8)}$ = 1.221 and p = 0.257). (g) Sucrose preference test: preference index ($t_{(5)}$ = 0.123 and p = 0.907). (H) Total grooming time in the open field test ($t_{(8)}$ = 0.124 and p = 0.905). Different cohorts of mice were used in (b-f, h) and (g). Data are expressed as mean ± SEM. Student's two-tailed unpaired t test. P values in (b-d) are adjusted by the Bonferroni correction. ns, not significant. Source data are provided in the Source Data file.

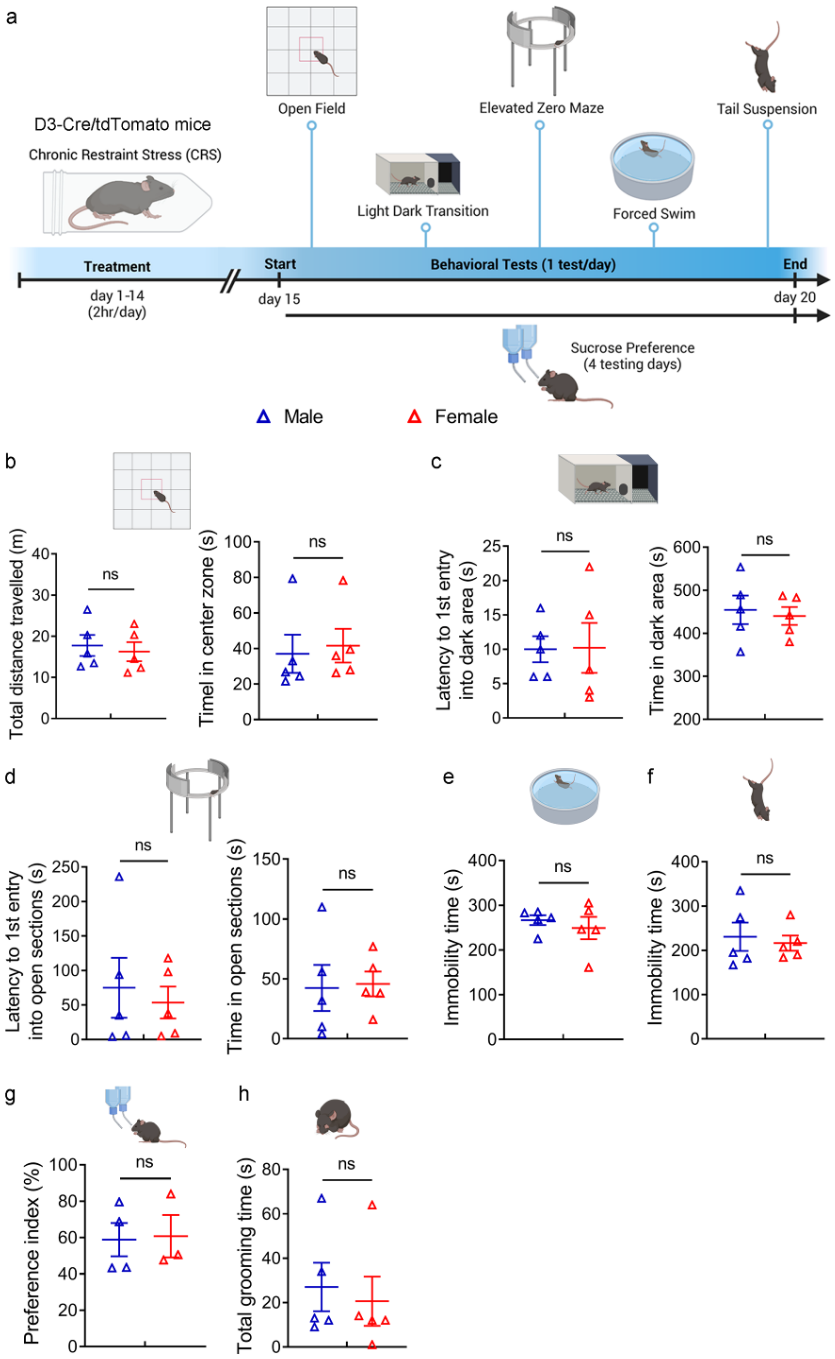

Supplementary Figure 2. Chronic restraint stress (CRS) does not induce sexually dimorphic effects on affective behaviors in D3-Cre/tdTomato mice. Related to Figure 1. (a) Experimental strategy and timeline for behavioral assays. Created with BioRender.com. Created with BioRender.com. (b-h) Effects of CRS on behavioral performance in different tests. (b) Open field test: total distance travelled (left; $t_{(8)} = 0.433$ and $p = 1.000$) and time in the center zone (right; $t_{(8)} = 0.318$ and $p = 1.000$). (c) Light-dark box transition test: latency to the first entry into the dark area (left; $t_{(8)} = 0.049$ and $p = 1.000$) and time spent in the dark area (right; $t_{(8)} = 0.367$ and $p = 1.000$). (d) Elevated zero maze test: latency to the first entry into open sections (left; $t_{(8)} = 0.435$ and $p = 1.000$) and time in open sections (right; $t_{(8)} = 0.156$ and $p = 1.000$). (e) Forced swimming test: immobility time ($t_{(8)} = 0.647$ and $p = 0.536$). (f) Tail suspension test: immobility time ($t_{(8)} = 0.397$ and $p = 0.702$). (g) Sucrose preference test: preference index ($t_{(5)} = 0.132$ and $p = 0.900$). (h) Total grooming time in the open field test ($t_{(8)} = 0.412$ and $p = 0.692$). Different cohorts of mice were used in (b-f, h) and (g). Data are expressed as mean ± SEM. Student's two-tailed unpaired t test. P values in (b)-(d) are adjusted by the Bonferroni correction. ns, not significant. Source data are provided in the Source Data file.

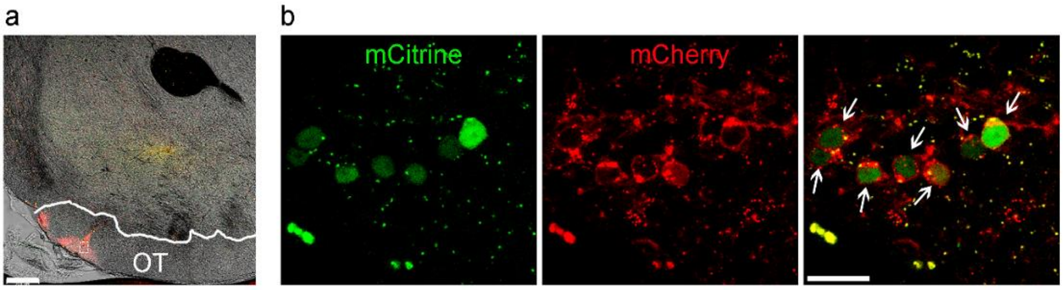

Supplementary Figure 3. Expression of excitatory and inhibitory DREADDs in OT D3 neurons. Related to Figures 4, 6. (a) Representative image from a coronal section showing the expression of hM$_3$D(Gq)-mCherry and KORD-mCitrine in IC D3 neurons in the OT. Scale bar = 200 µm. The expression of mCherry and mCitrine in the OT was confirmed postmortem for all mice included in the DREADD experiments. Only those with the right targets were included in further analysis. (b) Enlarged view of the rectangle area in (a) showing individual IC D3 neurons. Arrows denote neurons co-expressing hM$_3$D(Gq)-mCherry and KORD-mCitrine. Scale bar = 20 µm.

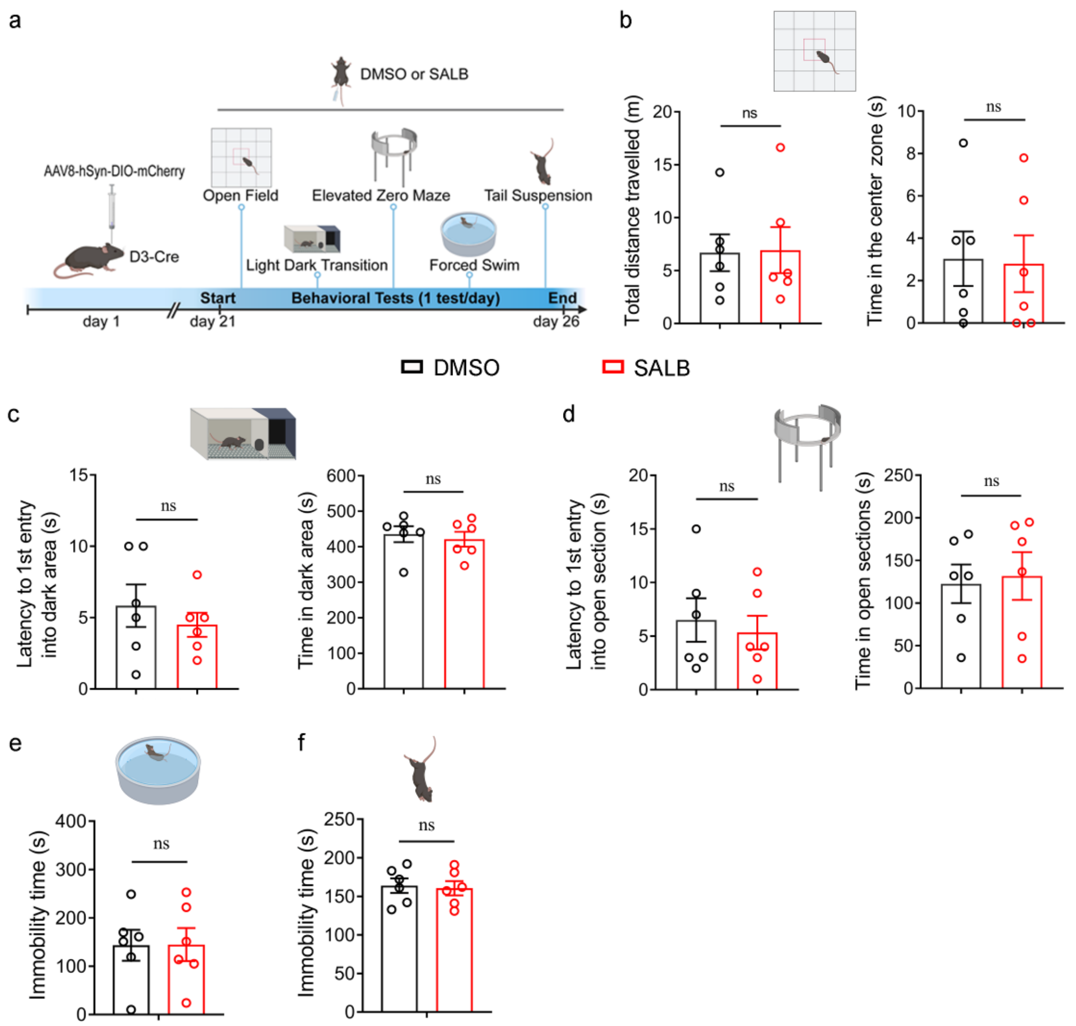

Supplementary Figure 4. D3-Cre mice with control virus (AAV8-DIO-mCherry) in OT D3 neurons do not change affective behaviors when treated with SALB. Related to Figure 4. (a) Schematic showing strategy for viral injection into the OT and timeline for behavioral assays. Created with BioRender.com. (b-f) Comparison of behavioral performance between DMSO- and SALB-treated mice. (b) Open field test: total distance travelled (left; $t_{(10)} = 0.091$ and $p = 1.000$) and time in the center zone (right; $t_{(10)} = 0.126$ and $p = 1.000$). (c) Light-dark box transition test: latency to the first entry into the dark area (left; $t_{(10)} = 0.777$ and $p = 1.000$) and time in the dark area (right; $t_{(10)} = 0.400$ and $p = 1.000$). (d) Elevated zero maze test: latency to the first entry into open sections (left; $t_{(10)} = 0.455$ and $p = 1.000$) and time in open sections (right; $t_{(10)} = 0.255$ and $p = 1.000$). (e) Forced swimming test: immobility time ($t_{(10)} = 0.032$ and $p = 0.975$). (f) Tail suspension test: immobility time ($t_{(10)} = 0.251$ and $p = 0.807$). n = 6 mice per group. Data are expressed as mean±SEM. Student's two-tailed unpaired t tests. P values in (b)-(d) are adjusted by the Bonferroni correction. ns, not significant. DMSO, dimethyl sulfoxide; SALB, salvinorin B. Source data are provided in the Source Data file.

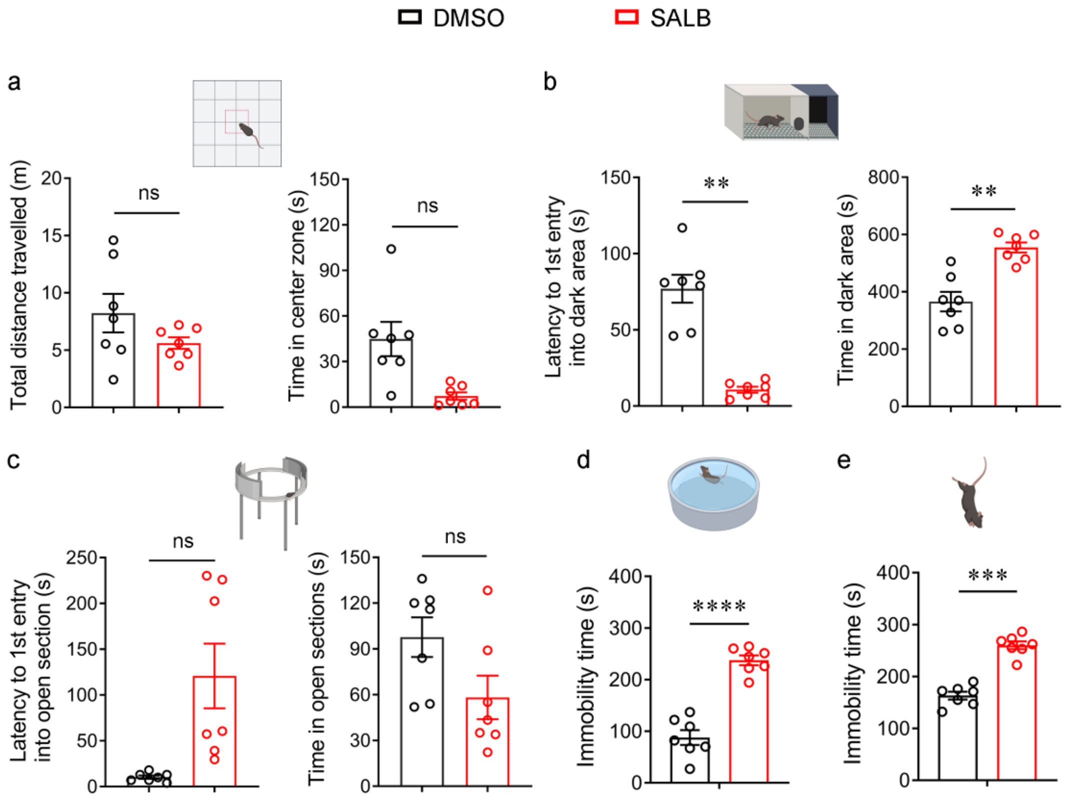

Supplementary Figure 5. Chemogenetic inhibition of OT D3 neurons changes affective behaviors in mice after rectification of decreased grooming. Related to Figure 4. (a-e) Effects of inhibition of D3 neurons on behavioral performance in different tests. (a) Open field test: total distance travelled (left; $t_{(6)}$ = 2.050 and p = 0.516) and time in the center zone (right; $t_{(6)}$ = 2.899 and p = 0.162). (b) Light-dark box transition test: latency to the first entry into the dark area (left; $t_{(6)}$ = 7.722 and p = 1.49×10^{-3}) and time in the dark area (right; $t_{(6)}$ = 5.541 and p = 0.009). (c) Elevated zero maze test: latency to the first entry into open sections (left; two-sided Wilcoxon matched-pairs signed rank test; p = 0.096) and time in open sections (right; $t_{(6)}$ = 2.243 and p = 0.396). (d) Forced swimming test: immobility time ($t_{(6)}$ = 11.98 and p = 2.05×10^{-5}). (e) Tail suspension test: immobility time ($t_{(6)}$ = 8.624 and p = 1.34×10^{-4}). n = 7 mice per group. Data are expressed as mean ± SEM. Student's two-tailed paired t tests. P values in (a)-(c) are adjusted by the Bonferroni correction. **p<0.01, ***p <0.001, ****p<0.0001; ns, not significant. DMSO, dimethyl sulfoxide; SALB, salvinorin B. Source data are provided in the Source Data file. Illustrations of behavioral tests are created with BioRender.com.

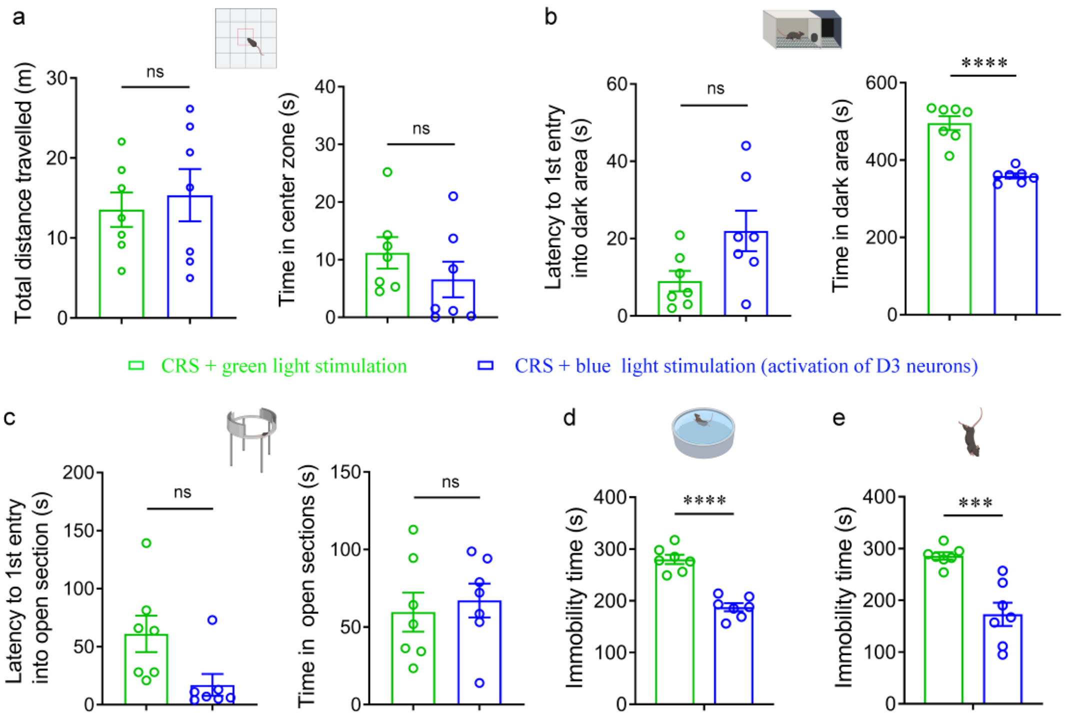

Supplementary Figure 6. Optogenetic activation of OT D3 neurons reverses CRS-induced depression-like behaviors after rectification of increased grooming. Related to Figure 5. (a-e) Effects of blue light activation of D3 neurons on behavioral performance of CRS-treated mice in different tests compared to green light stimulation. (a) Open field test: total distance travelled (left; $t_{(12)}$ = 0.467 and p = 1.000) and time in the center zone (right; $t_{(12)}$ = 1.120 and p = 1.000). (b) Light-dark box transition test: latency to the first entry into the dark area (left; $t_{(12)}$ = 2.220 and p = 0.276) and time in the dark area (right; $t_{(12)}$ = 7.136 and p = 0.71×10^{-4}). (c) Elevated zero maze test: latency to the first entry into open sections (left; two-sided Mann Whitney test; p = 0.059) and time in open sections (right; $t_{(12)}$ = 0.450 and p = 3.966). (d) Forced swimming test: immobility time ($t_{(12)}$ = 7.883 and p = 4.37×10^{-6}). (e) Tail suspension test: immobility time ($t_{(12)}$ = 4.777 and p = 4.51×10^{-4}). n = 7 mice per group. Data are expressed as mean ± SEM. Student's two-tailed unpaired t tests. P values in (a)-(c) are adjusted by the Bonferroni correction. ***p<0.001, ****p<0.0001; ns, not significant. Source data are provided in the Source Data file. Illustrations of behavioral tests are created with BioRender.com.

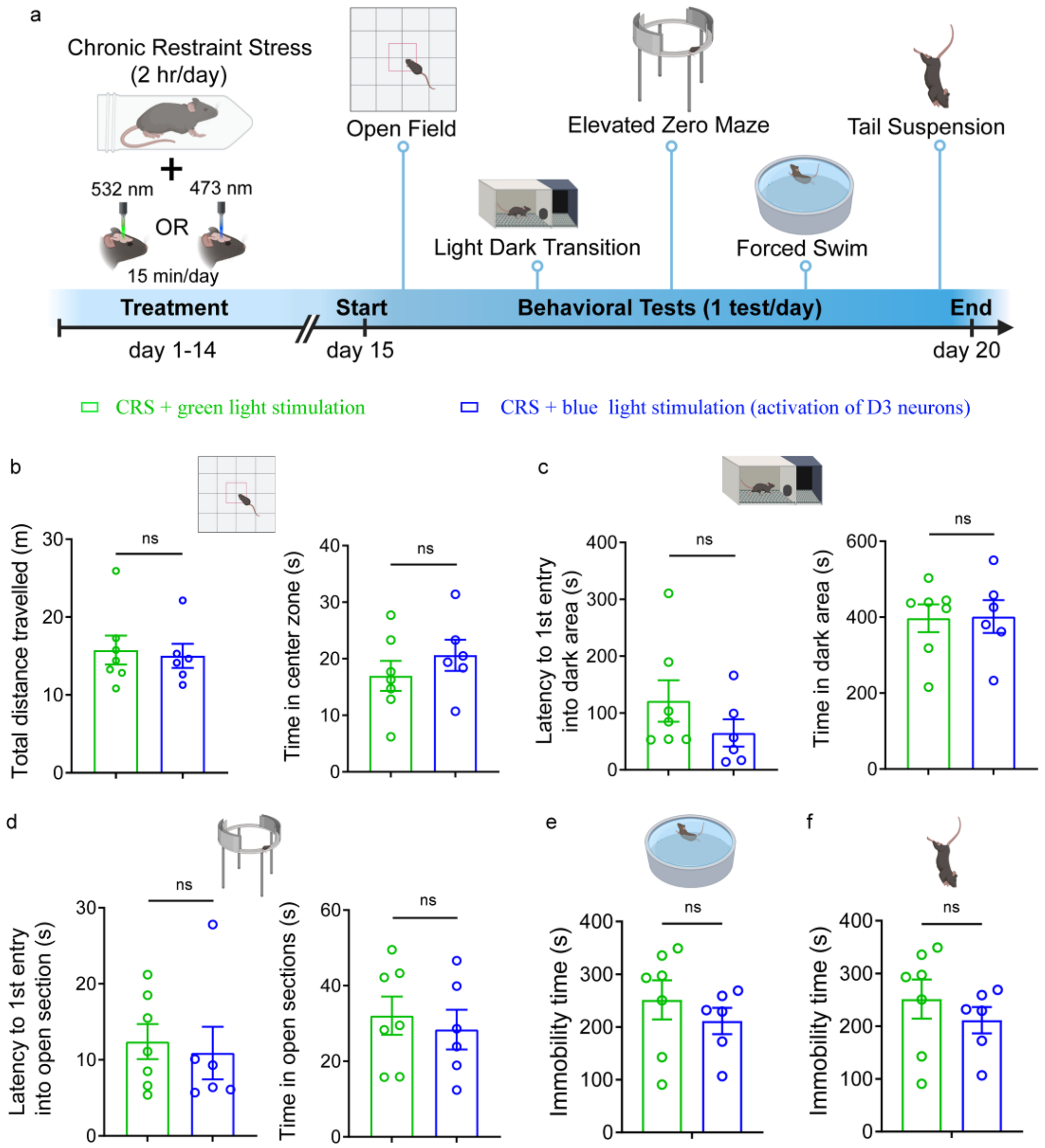

Supplementary Figure 7. Optogenetic activation of OT D3 neurons after each CRS session alone does not normalize CRS-induced depression-like behaviors. Related to Figure 5. (a) Experimental strategy and timeline of behavioral assays. (b-f) Effects of blue light activation of D3 neurons on behavioral performance of CRS mice compared to green light stimulation. (b) Open field test: total distance travelled (left; $t_{(11)} = 0.299$ and $p = 1.000$) and time in the center zone (right; $t_{(11)} = 0.953$ and $p = 1.000$). (c) Light-dark box transition test: latency to the first entry into the dark area (left; $t_{(11)} = 1.242$ and $p = 1.000$) and time in the dark area (right; $t_{(11)} = 0.076$ and $p = 1.000$). (d) Elevated zero maze test: latency to the first entry into open sections (left; two-sided Mann-Whitney test; $p = 1.000$) and time in open sections (right; $t_{(11)} = 0.503$ and $p = 1.000$). (e) Forced swimming test: immobility time ($t_{(11)} = 0.861$ and $p = 0.408$). (f) Tail suspension test: immobility time ($t_{(11)} = 1.587$ and $p = 0.141$). CRS+ green light stimulation: n = 7 mice; CRS+ blue light stimulation: n = 6 mice. Data are expressed as mean ± SEM. Student's two-tailed unpaired t tests. P values in (b)-(d) are adjusted by the Bonferroni correction. ns, not significant. Source data are provided in the Source Data file. Illustrations of behavioral tests are created with BioRender.com.

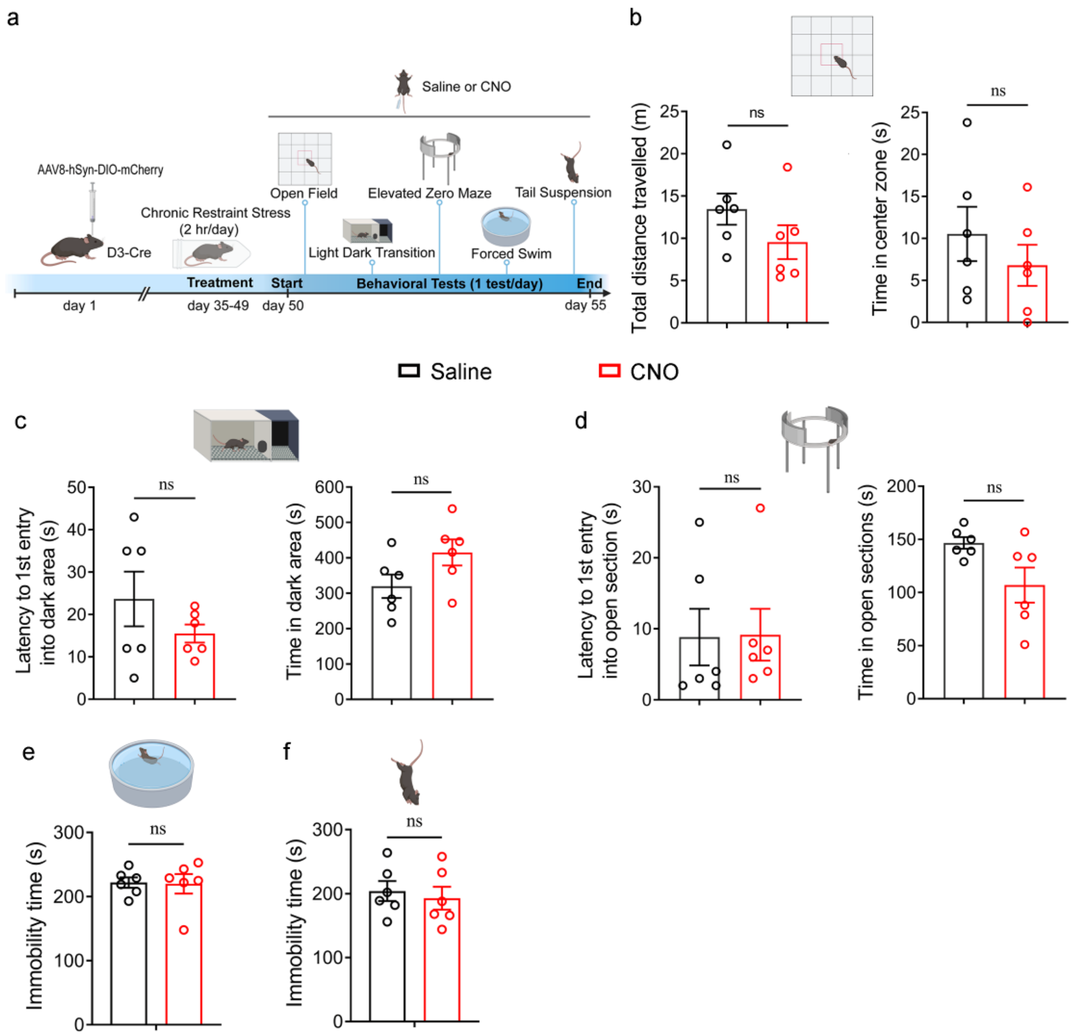

Supplementary Figure 8. CNO treatment of D3-Cre mice with control virus (AAV8-DIO-mCherry) in OT D3 neurons does not ameliorate CRS-induced behavioral changes. Related to Figure 6. (a) Schematic showing strategy for viral injection into the OT and timeline for behavioral assays. Created with BioRender.com. (b-f) Comparison of behavioral performance between saline- and CNO-treated mice. (b) Open field test: total distance travelled (left; $t_{(10)} = 1.430$ and $p = 1.000$) and time in the center zone (right; $t_{(10)} = 0.920$ and $p = 1.000$). (c) Light-dark box transition test: latency to the first entry into the dark area (left; $t_{(10)} = 1.201$ and $p = 1.000$) and time in the dark area (right; $t_{(10)} = 1.919$ and $p = 0.504$). (d) Elevated zero maze test: latency to the first entry into open sections (left) and time in open sections (right) ($p = 1.000$ and 0.558, respectively). (e) Forced swimming test: immobility time ($p = 0.729$). (f) Tail suspension test: immobility time ($t_{(10)} = 0.470$ and $p = 0.649$). n = 6 mice per group. Data are expressed as mean ± SEM. Student's two-tailed unpaired t tests were used except for (d) and (e) in which two-sided Mann-Whitney test were used. P values in (b)-(d) are adjusted by the Bonferroni correction. ns, not significant. CNO, Clozapine N-oxide. Source data are provided in the Source Data file.

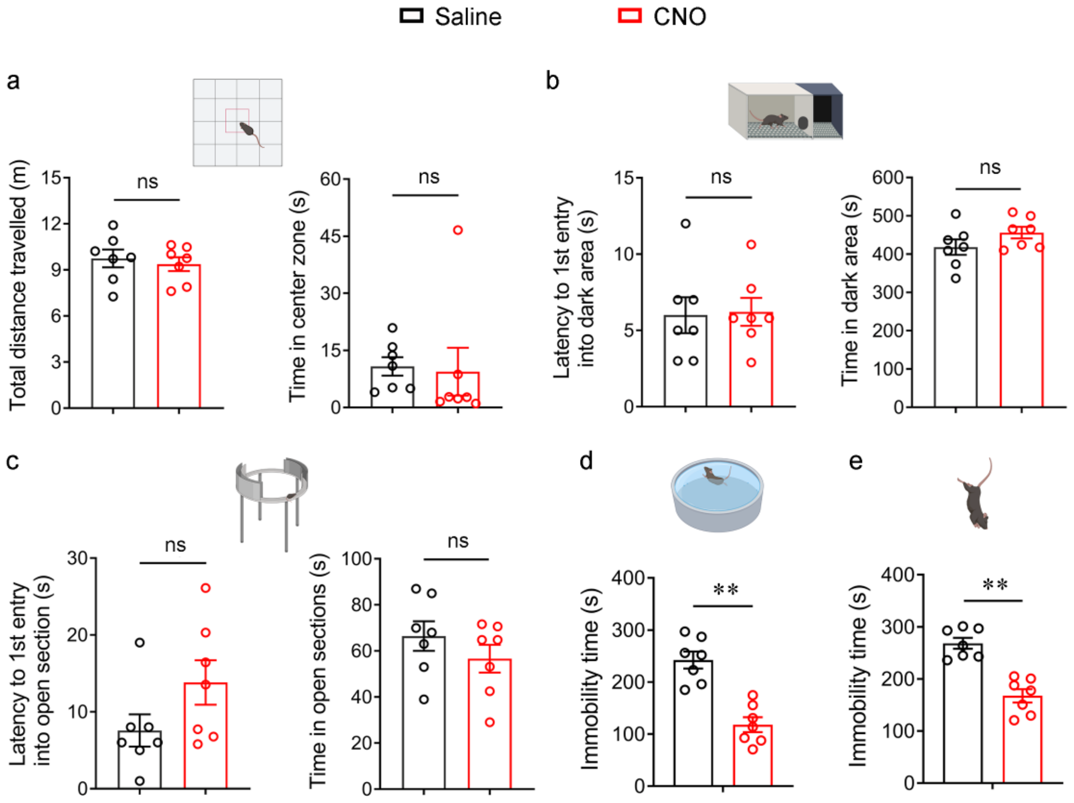

Supplementary Figure 9. Chemogenetic activation of OT D3 neurons normalizes CRS-induced depression-like behaviors after rectification of increased grooming. Related to Figure 6. (a-e) Effects of chemogenetic activation of D3 neurons on behavioral performance in different tests. (a) Open field test: total distance travelled (left; $t_{(6)} = 0.465$ and $p = 1.000$) and time in the center zone (right; two-sided Wilcoxon matched-pairs signed rank test; $p = 1.000$). (b) Light-dark box transition test: latency to the first entry into the dark area (left; $t_{(6)} = 0.170$ and $p = 1.000$) and time in the dark area (right; $t_{(6)} = 1.304$ and $p = 1.000$). (c) Elevated zero maze test: latency to the first entry into open sections (left; $t_{(6)} = 1.849$ and $p = 0.684$) and time in open sections (right; $t_{(6)} = 1.408$ and $p = 1.000$). (d) Forced swimming test: immobility time ($t_{(6)} = 5.240$ and $p = 0.0019$). (e) Tail suspension test: immobility time ($t_{(6)} = 5.034$ and $p = 0.0024$). n = 7 mice per group. Student's two-tailed paired t tests. Data are expressed as mean ± SEM. P values in (a)-(c) are adjusted by the Bonferroni correction. **$p<0.01$; ns, not significant. CNO, Clozapine N-oxide. Source data are provided in the Source Data file. Illustrations of behavioral tests are created with BioRender.com.
