# Supplementary material for: Ventral striatal islands of Calleja neurons bidirectionally mediate depression-like behaviors in mice
Source: Nat Commun. 2023 Oct 28;14:6887. doi: 10.1038/s41467-023-42662-z (PMC10613228; doi:10.1038/s41467-023-42662-z)
Supplement: Supplementary file 3 — Reporting Summary [file 41467_2023_42662_MOESM3_ESM.pdf]

nature portfolio

Corresponding author(s): Yun-Feng Zhang and Minghong Ma

Last updated by author(s): 10/04/2023

Reporting Summary

Nature Portfolio wishes to improve the reproducibility of the work that we publish. This form provides structure for consistency and transparency in reporting. For further information on Nature Portfolio policies, see our Editorial Policies and the Editorial Policy Checklist.

Statistics

For all statistical analyses, confirm that the following items are present in the figure legend, table legend, main text, or Methods section.

n/a	Confirmed	
☐	☒	The exact sample size (n) for each experimental group/condition, given as a discrete number and unit of measurement
☐	☒	A statement on whether measurements were taken from distinct samples or whether the same sample was measured repeatedly
☐	☒	The statistical test(s) used AND whether they are one- or two-sided<br>*Only common tests should be described solely by name; describe more complex techniques in the Methods section.*
☐	☒	A description of all covariates tested
☐	☒	A description of any assumptions or corrections, such as tests of normality and adjustment for multiple comparisons
☐	☒	A full description of the statistical parameters including central tendency (e.g. means) or other basic estimates (e.g. regression coefficient) AND variation (e.g. standard deviation) or associated estimates of uncertainty (e.g. confidence intervals)
☐	☒	For null hypothesis testing, the test statistic (e.g. F, t, r) with confidence intervals, effect sizes, degrees of freedom and P value noted<br>*Give P values as exact values whenever suitable.*
☒	☐	For Bayesian analysis, information on the choice of priors and Markov chain Monte Carlo settings
☒	☐	For hierarchical and complex designs, identification of the appropriate level for tests and full reporting of outcomes
☒	☐	Estimates of effect sizes (e.g. Cohen's d, Pearson's r), indicating how they were calculated

Our web collection on statistics for biologists contains articles on many of the points above.

Software and code

Policy information about availability of computer code

Data collection	Commercial software used to collect the data in this study include LAS AF Lite and PULSE v8.74.
Data analysis	Commercial software used to analyze the data in this study include Igor Pro 6, Prism 7, and ANY-maze 6.35.

For manuscripts utilizing custom algorithms or software that are central to the research but not yet described in published literature, software must be made available to editors and reviewers. We strongly encourage code deposition in a community repository (e.g. GitHub). See the Nature Portfolio guidelines for submitting code & software for further information.

Data

Policy information about availability of data

All manuscripts must include a data availability statement. This statement should provide the following information, where applicable:

- Accession codes, unique identifiers, or web links for publicly available datasets
- A description of any restrictions on data availability
- For clinical datasets or third party data, please ensure that the statement adheres to our policy

Due to multiple specialized platforms (including HEKA PULSE software, Igor Pro, ANY-maze, and LAS AF Lite) used to collect and/or analyze the data and the enormous size of the raw data (confocal images, behavioral videos, and ex vivo patch clamp recordings), it is not feasible for us to make them publicly accessible. However, we will make the raw data available to readers upon request. Source data for all graphs are provided with this paper.

Human research participants

Policy information about studies involving human research participants and Sex and Gender in Research.

Reporting on sex and gender	N/A
Population characteristics	N/A
Recruitment	N/A
Ethics oversight	N/A

Note that full information on the approval of the study protocol must also be provided in the manuscript.

Field-specific reporting

Please select the one below that is the best fit for your research. If you are not sure, read the appropriate sections before making your selection.

[x] Life sciences [ ] Behavioural & social sciences [ ] Ecological, evolutionary & environmental sciences

For a reference copy of the document with all sections, see nature.com/documents/nr-reporting-summary-flat.pdf

Life sciences study design

All studies must disclose on these points even when the disclosure is negative.

Sample size	Sample sizes for individual experiments were determined according to the NIH "Guidelines for the Care and Use of Mammals in Neuroscience and Behavioral Research" (Sample Size Determination: https://www.ncbi.nlm.nih.gov/books/NBK43321/).
Data exclusions	No data were excluded unless the viral injection was off target.
Replication	Various controls (genotype, virus, laser wavelength, etc) were included in experimental design. The number of replications for each experiment was included in the figure legends.
Randomization	Animals were allocated randomly in the experiments and various stimulating conditions (e.g., blue versus green laser) for individual mice were randomized unless otherwise stated.
Blinding	Blinding in data collection and analysis were carried out as much as possible.

Reporting for specific materials, systems and methods

We require information from authors about some types of materials, experimental systems and methods used in many studies. Here, indicate whether each material, system or method listed is relevant to your study. If you are not sure if a list item applies to your research, read the appropriate section before selecting a response.

Materials & experimental systems

n/a	Involved in the study	
[ ]	[x]	Antibodies
[x]	[ ]	Eukaryotic cell lines
[x]	[ ]	Palaeontology and archaeology
[ ]	[x]	Animals and other organisms
[x]	[ ]	Clinical data
[x]	[ ]	Dual use research of concern

Methods

n/a	Involved in the study	
[x]	[ ]	ChIP-seq
[x]	[ ]	Flow cytometry
[x]	[ ]	MRI-based neuroimaging

Antibodies

Antibodies used	Primary anti tyrosine hydroxylase (TH) antibody (1:500; Millipore, cat# AB152, host: rabbit), and the secondary antibody (1:1000; Life Technology, cat# A31573, host: donkey)
Validation	validation from the manufacturer's website<br>https://www.emdmillipore.com/US/en/product/Anti-Tyrosine-Hydroxylase-Antibody,MM_NF-AB152#anchor_BRO

Animals and other research organisms

Policy information about studies involving animals; ARRIVE guidelines recommended for reporting animal research, and Sex and Gender in Research

Laboratory animals	The D1-tdTomato and D2-EGFP (Tg(Drd2-EGFP)S118Gsat) mice were crossed to obtain double-transgenic mice in which dopamine D1 and D2 receptor-expressing neurons are labeled with red and green fluorescence, respectively. Bacterial artificial chromosome (BAC) transgenic D3-Cre (Tg(Drd3-cre)KI198Gsat) was obtained from Mutant Mouse Resource & Research Centers (MMRRC) and crossed with the Cre-dependent tdTomato reporter line (JAX Stock No: 007909 or Ai9 line: Rosa26-floxed-tdTomato) or Cre-dependent channel rhodopsin 2 (ChR2) line (JAX Stock No: 024109 or Ai32 line: Rosa26-floxed-ChR2) to generate D3-Cre/tdTomato or D3-Cre/ChR2 mice, respectively. This D3-Cre line was chosen because Cre expression is more restricted to the islands of Calleja neurons. Drd1-Cre (D1-Cre for simplicity; MMRRC Strain# 37156-Jax) mice were obtained from MMRRC. Both male and female mice (8-12 weeks old) were used in all experiments.
Wild animals	No wild animals were used in the study
Reporting on sex	Both male and female mice were used in all experiments and they were separately analyzed initially (Supplementary Fig. 1 and supplementary Fig. 2). Since no sex difference was evident, in later experiments, the data from both sexes were pooled.
Field-collected samples	No field collected samples were used in the study.
Ethics oversight	All animal procedures were in compliance with the governmental regulations in China and USA and approved by the Institutional Animal Care and Use Committee of the Institute of Zoology, Chinese Academy of Sciences, of the University of Pennsylvania, and of the University of Florida.

Note that full information on the approval of the study protocol must also be provided in the manuscript.
